# Analysis of the Effect of Intestinal Ischemia and Reperfusion on the Rat Neutrophils Proteome

**DOI:** 10.3389/fmolb.2018.00089

**Published:** 2018-11-29

**Authors:** Muhammad Tahir, Samina Arshid, Belchor Fontes, Mariana S. Castro, Isabelle S. Luz, Katyelle L. R. Botelho, Simone Sidoli, Veit Schwämmle, Peter Roepstorff, Wagner Fontes

**Affiliations:** ^1^Laboratory of Biochemistry and Protein Chemistry, Department of Cell Biology, Institute of Biology, University of Brasilia, Brasília, Brazil; ^2^Department of Biochemistry and Molecular Biology, University of Southern Denmark, Odense, Denmark; ^3^Laboratory of Surgical Physiopathology (LIM-62), Faculty of Medicine, University of São Paulo, São Paulo, Brazil

**Keywords:** ischemia reperfusion, neutrophils, proteomics, systemic inflammatory response, LC-MS/MS

## Abstract

Intestinal ischemia and reperfusion injury is a model system of possible consequences of severe trauma and surgery, which might result into tissue dysfunction and organ failure. Neutrophils contribute to the injuries preceded by ischemia and reperfusion. However, the mechanisms by which intestinal ischemia and reperfusion stimulate and activate circulating neutrophils is still not clear. In this work, we used proteomics approach to explore the underlying regulated mechanisms in Wistar rat neutrophils after ischemia and reperfusion. We isolated neutrophils from three different biological groups; control, sham laparotomy, and intestinal ischemia/reperfusion. In the workflow, we included iTRAQ-labeling quantification and peptide fractionation using HILIC prior to LC-MS/MS analysis. From proteomic analysis, we identified 2,045 proteins in total that were grouped into five different clusters based on their regulation trend between the experimental groups. A total of 417 proteins were found as significantly regulated in at least one of the analyzed conditions. Interestingly, the enzyme prediction analysis revealed that ischemia/reperfusion significantly reduced the relative abundance of most of the antioxidant and pro-survival molecules to cause more tissue damage and ROS production whereas some of the significantly up regulated enzymes were involved in cytoskeletal rearrangement, adhesion and migration. Clusters based KEGG pathways analysis revealed high motility, phagocytosis, directional migration, and activation of the cytoskeletal machinery in neutrophils after ischemia and reperfusion. Increased ROS production and decreased phagocytosis were experimentally validated by microscopy assays. Taken together, our findings provide a characterization of the rat neutrophil response to intestinal ischemia and reperfusion and the possible mechanisms involved in the tissue injury by neutrophils after intestinal ischemia and reperfusion.

## Introduction

The intestine is the most sensitive organ to ischemia and reperfusion (IR) injury. This injury can result from various clinical situations, such as intestinal obstruction, acute mesenteric ischemia, incarcerated hernia, small intestine transplantation, neonatal necrotizing enterocolitis, trauma, and shock, taking the patient to relentless clinical syndromes, and even death (Mojzis et al., 2001; Mallick et al., 2004; Guneli et al., 2007). It is clear now that the reperfusion following ischemia leads to significantly greater mucosal intestinal injury as compared to the ischemia alone (Crissinger and Granger, 1989) whereas development of the systemic inflammatory response syndrome (SIRS) and multiple organ failure (MOF) can be the final consequences of IR (Ceppa et al., 2003).

Among the polymorphonuclear leukocytes (PMNs), neutrophils are the first line of defense against bacterial and fungal infections (Kaufmann, 2008). However, a large number of studies showed that IR injury is mainly because of PMN and endothelial cell (EC) interactions in reperfused tissues (Massberg et al., 1998; Kumar et al., 2009; Kvietys and Granger, 2012). Normally, a multistep process of neutrophil recruitment to the site of infection requires three types of adhesion receptors, like integrins, selectins, and adhesion receptors of the immunoglobulin superfamily (Rao et al., 2007).

It has been shown that P- and E-selectins are over expressed in a mouse model after intestinal ischemia/reperfusion and that inhibiting P-selectin decreased neutrophil rolling and adhesion, reducing the injury (Riaz et al., 2002). Another IR model showed decreased neutrophil rolling and adherence by blocking of P- and L-selectins (Kubes et al., 1995). The up-regulation of adhesion molecules on the endothelial surface was observed following ischemia/reperfusion injury (IRI) that can result in diapedesis of neutrophils, further contributing to muscle dysfunction (Hierholzer et al., 1999). Similarly, in small intestine, an increase in expression of inflammatory mediators like tumor necrosis factor-α (TNF-α), cyclooxygenase-2 (COX-2) and intercellular adhesion molecule-1 (ICAM-1), have been observed after IR with increase in neutrophil infiltration (Watanabe et al., 2014). Another study demonstrated systemic serum level elevation of the CC chemokines along with XC chemokines in a model of intestinal ischemia, hence leading to greater PMNs activation and tissue injury (Jawa et al., 2013). Each of these signals interact with specific receptors expressed on the plasma membrane of PMNs with an overlapping array of signal transduction pathways leading to functional responses such as rearrangement of the actin cytoskeleton (Luerman et al., 2010).

A gradient of chemoattractant signals arising from the dying tissues helps in the recruitment of PMNs (McDonald et al., 2010) that secrete a large number of factors like reactive oxygen species, chemokines, cytokines, lipid mediators, and proteases (Rodrigues and Granger, 2010). However, molecular mechanisms, enzymes, and pathways by which PMNs participate in the IR injury are not fully understood. The current understanding of the interconnections among the many signal transduction pathways that regulate neutrophil activation is incomplete. Proteomics research has improved the understanding of the neutrophil biology in the past, and few publications describe the effect of the inflammatory response on the PMNs proteome (Morris et al., 2008). There is one proteomic study on the response to intestinal ischemia and reperfusion, however limited on molecules expressed by the intestinal epithelium, and there is lack of understanding regarding the PMNs response against IR. The main objective of this study is to explore the effect of IR on the PMNs in rats using mass spectrometry based proteomics (Hurst et al., 1998). We analyzed the neutrophil proteome from three different biological conditions, including control, sham laparotomy and intestinal ischemia/reperfusion. Proteomic analysis of neutrophils after ischemia and reperfusion revealed that neutrophils down regulate the expression of different antioxidant, pro-survival molecules. Significantly up regulated oxidoreductases and down regulated transferases can interfere in the integrin signaling pathway, lipid metabolism and reactive oxygen species (ROS) generation, leading to the local and remote tissues injury. Furthermore, our analysis shows the regulation of different proteins and pathways required for neutrophil adhesion, directional migration, and phagocytosis after intestinal ischemia and reperfusion. Down regulation of important enzymes from LTB4 synthesis pathway opens some questions that need further analysis. Functional assays revealing increased ROS production and decreased phagocytosis after ischemia/reperfusion are coherent with the proteomics findings. We anticipate that these findings will provide trustworthy basis for further deep analysis in neutrophil biology.

## Results

### Protein identification and relative abundance profile

**Figure 1:**
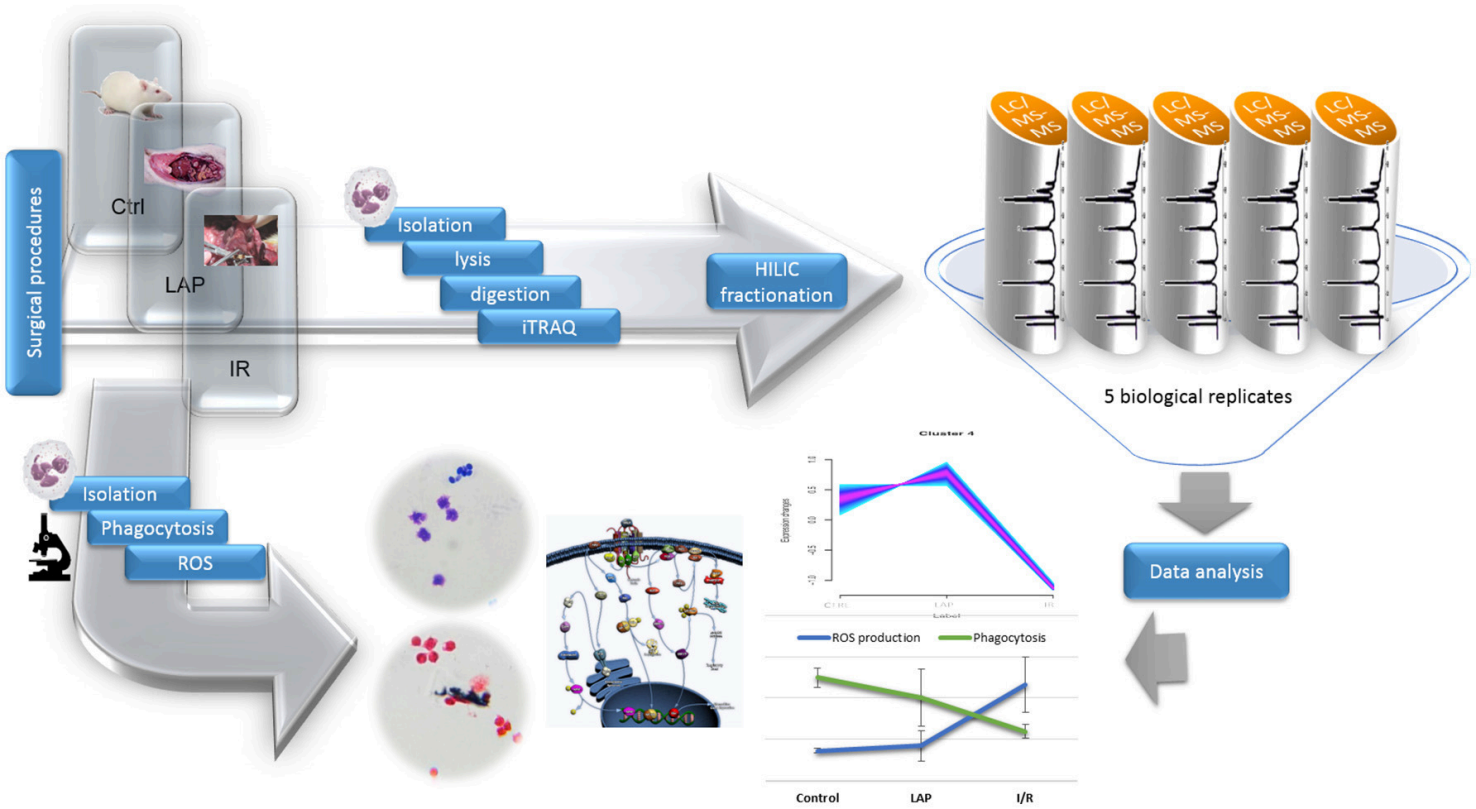
Experimental design. Surgical procedures were followed by isolation of neutrophils from the three conditions. Protein extraction was performed by using FASP protocol. After Hilic fractionations, LTQ Orbitrap MS analysis was performed and the data obtained was analyzed by using bioinformatics tools. Another set of isolated neutrophils was used to determine the phagocytosis rate and ROS production by optical microscopy.

For the large-scale proteomics analysis of rat neutrophils, samples were collected from three experimental groups (Figure 1) including control, laparotomy and intestinal ischemia/reperfusion groups. Proteins from each experimental group with five biological replicates were iTRAQ labeled for relative quantification analysis. To find the significant changes in protein abundance among three groups, unique peptides with high confidence identification and their respective iTRAQ reporter ion intensity values were analyzed by using R. For a detailed description of the methods, see the Supplementary Methods section. Mass spectrometry analysis resulted in the identification of 2045 proteins (Supplementary Table S1).

**Figure 2:**
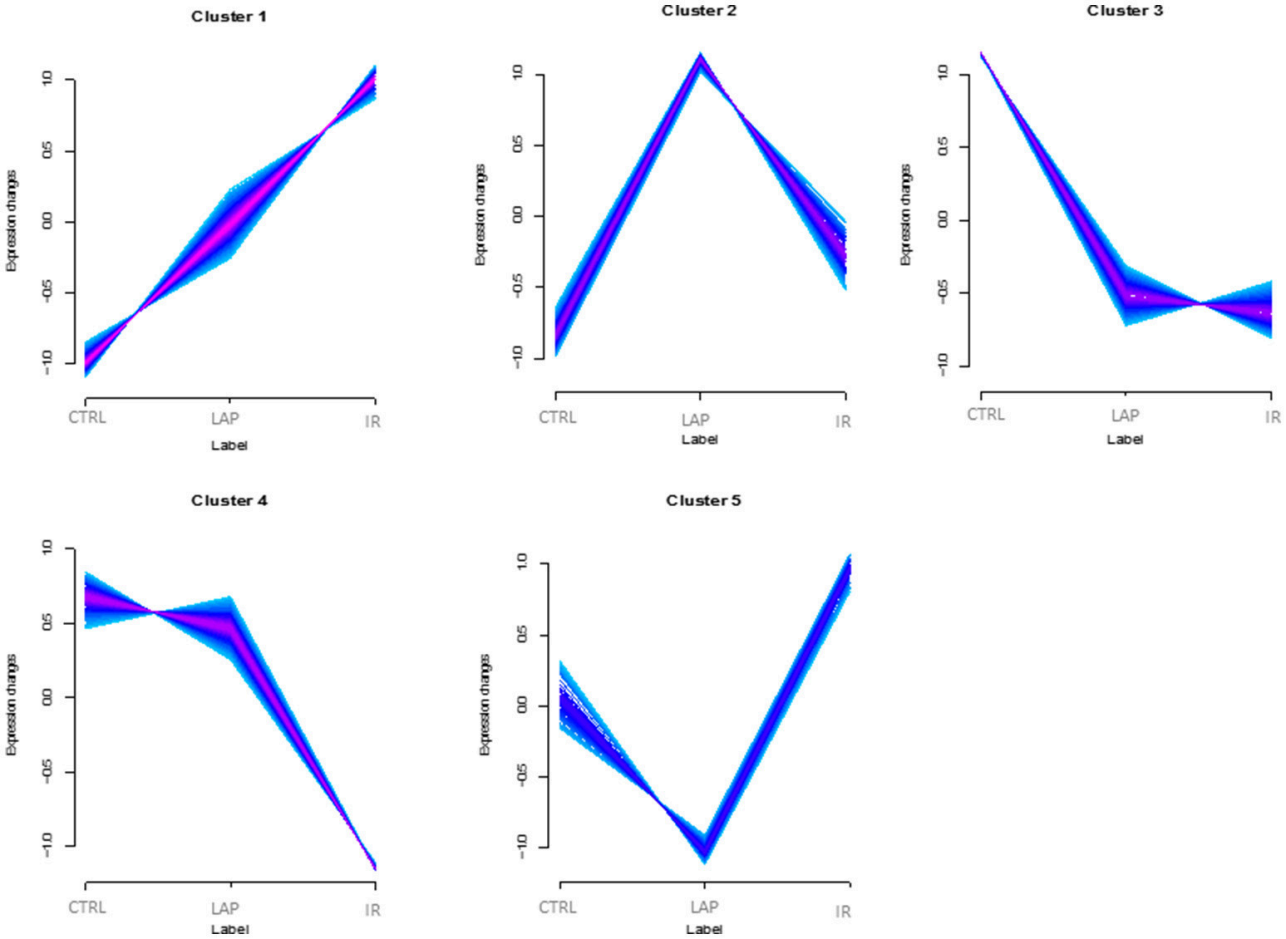
Abundance profiles of rat neutrophils proteins. Abundance profile of the regulated proteins in control (Ctrl), laparotomy (LAP), and ischemia/reperfusion (IR) experimental groups. The x-axis shows the different conditions and y-axis represents expression change (*z*-scores). The colors correspond to the membership values, representing the degree of how much a protein belongs to the nearest cluster.

After the data analysis, proteins were grouped into clusters according to their abundance profile among the three conditions. To assign the proteins according to their abundance in the best number of clusters, two validation indices, Xie-Beni index (Xie and Beni, 1991) and minimal centroid distance (Schwammle and Jensen, 2010), were applied and proteins were assigned to five different clusters based on their regulation trend (Figure 2, Supplementary Table S2). Expression changes are shown as z scores (i.e., relative abundances normalized by mean and standardized by dividing by the standard deviation). The colors correspond to the so-called membership values (values in the range [0.1]), representing the degree of how much a protein belongs to the nearest cluster. Only proteins with memberships above 0.5 are shown. A subset of 417 proteins was significantly regulated (Limma and rank products *q*-value < 0.05) in at least one of the analyzed conditions within the five clusters. A standard PCA analysis was performed to check the similarity among different samples.

### Go *slim* analysis and enzyme activity prediction for total rat neutrophil proteome

**Figure 3:**
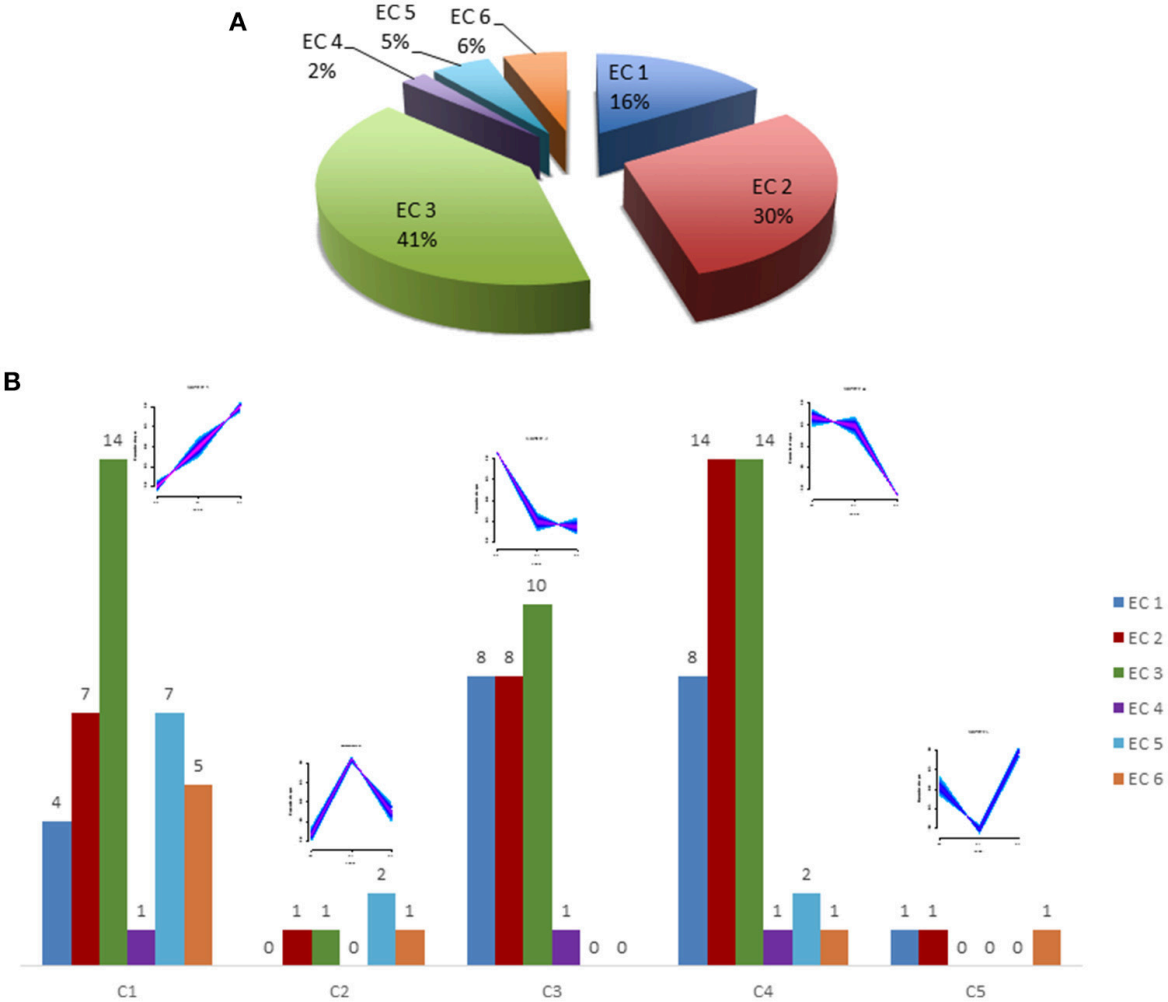
Enzyme activity prediction. **(A)** A pie chart shows the predicted enzyme activity of six classes of enzymes for total rat neutrophil proteome. **(B)** A bar chart shows predicted enzyme activity for regulated proteins from all 5 clusters. EC 1 represents Oxidoreductases, EC 2–Transferases, EC 3–Hydrolases, EC 4–Lyases, EC 5–Isomerases, and EC 6–Ligases. A detail of the predicted enzymatic activities of these proteins is listed in Table 1. The small images above each cluster show the general cluster pattern. For details on this see Figure 2.

Protein classification was performed by GO *slim* analysis using ProteinCenter (Thermo Scientific) as a platform. The resultant cellular component, biological process and molecular function terms for the five clusters are shown in Supplementary Figure S1. Molecular function analysis revealed that about 33% of the neutrophil proteome has predicted enzymatic activity. It is composed of 18% oxidoreductases (EC:1), 27% transferases (EC:2), 38% hydrolases (EC:3), 3% lyases (EC:4), 5% isomerases (EC:5) and 9% ligases (EC:6) as predicted activity, as shown in Figure 3A. The overall distribution of the enzymes across five clusters is illustrated in Figure 3B whereas Table 1 represents the significantly regulated proteins in at least two conditions among 5 clusters.

**Table 1:** Enzyme activities prediction for the significantly regulated proteins.

**Enzyme description**	**EC number**	**Protein IDs**	**Gene symbol**	**Cluster no**.	**LAP vs. Ctrl Log2 ratio**	**IR vs. Ctrl Log2 ratio**	**IR vs. LAP Log2 ratio**
**OXIDOREDUCTASES**							
superoxide dismutase [Cu-Zn]	EC 1.15.1.1	P07632	Sod1	1	0.108233771	0.130217203	0.021983432
Fatty acyl-CoA reductase 1 isoform 1	EC 1.2.1.n2	Q922J9	Far1	1	0.335933388	0.471008515	0.135075127
Prostaglandin reductase 1	EC 1.3.1.48	P97584	Ptgr1	1	0.151344167	0.63932175	0.487977582
Dihydrofolate reductase	EC 1.5.1.3	Q920D2	Dhfr	1	0.202589021	0.26928123	0.06669221
NAD-dependent malic enzyme, mitochondrial precursor	EC 1.1.1.38	Q99KE1	Me2	3	−0.125334931	−0.211535203	−0.086200272
Eosinophil peroxidase precursor	EC 1.11.1.7	P49290	Epx	3	−1.461573896	−1.86665744	−0.405083544
Glutathione peroxidase 1	EC 1.11.1.9	P04041	Gpx1	3	−0.131894129	−0.236200267	−0.104306138
Protein-methionine sulfoxide oxidase MICAL1	EC 1.14.13.-	D3ZBP4	Mical1	3	−0.132806847	−0.172661531	−0.039854685
Leukotriene-B(4) omega-hydroxylase 2	EC 1.14.13.30	Q3MID2	Cyp4f18	3	−0.314624374	−0.54501604	−0.230391666
Metalloreductase STEAP4	EC 1.16.1.-	Q4V8K1	Steap4	3	−0.634298418	−0.633934112	0.000364306
Peroxisomal acyl-coenzyme A oxidase 1	EC 1.3.3.6	P07872	Acox1	3	−0.153657032	−0.11360617	0.040050862
NADPH–cytochrome P450 reductase	EC 1.6.2.4	P00388	Por	3	−0.14668384	−0.203604695	−0.056920854
GDP-L-fucose synthase	EC 1.1.1.271	P23591	Tsta3	4	−0.053953695	−0.146615802	−0.092662107
Myeloperoxidase precursor	EC 1.11.2.2	P11247	Mpo	4	−0.010129062	−0.152429094	−0.142300032
Arachidonate 15-lipoxygenase	EC 1.13.11.33	Q02759	Alox15	4	−0.591830552	−1.210220167	−0.618389614
Xanthine dehydrogenase/oxidase	EC 1.17.1.4	P22985	Xdh	4	−0.049187813	−0.100438443	−0.05125063
Aldehyde dehydrogenase family 3 member B1	EC 1.2.1.5	Q5XI42	Aldh3b1	4	−0.044890506	−0.14087961	−0.095989104
acyl-CoA dehydrogenase family member 9, mitochondrial	EC 1.3.99.-	Q8JZN5	Acad9	4	0.094393882	−0.091932542	−0.186326425
NAD(P)H dehydrogenase [quinone] 1	EC 1.6.5.2	P05982	Nqo1	4	−0.094854334	−0.270101397	−0.175247063
Thioredoxin reductase 1, cytoplasmic	EC 1.8.1.9	O89049	Txnrd1	4	−0.001836217	−0.119040483	−0.117204265
L-lactate dehydrogenase C chain	EC 1.1.1.27	P19629	Ldhc	5	−0.156311009	0.132653839	0.288964848
**TRANSFERASES**							
Non-specific lipid-transfer protein	EC 2.3.1.176	P11915	Scp2	1	0.197153822	0.41885748	0.221703658
Glycylpeptide N-tetradecanoyltransferase 1	EC 2.3.1.97	O70310	Nmt1	1	0.054980313	0.204797239	0.149816926
Dolichyl-diphosphooligosaccharide–protein glycosyltransferase 48 kDa subunit precursor	EC 2.4.99.18	O54734	Ddost	1	0.093155294	0.086662	−0.006493294
Dolichyl-diphosphooligosaccharide–protein glycosyltransferase subunit 1 precursor	EC 2.4.99.18	P07153	Rpn1	1	0.058894484	0.175025097	0.116130613
Dolichyl-diphosphooligosaccharide–protein glycosyltransferase subunit DAD1	EC 2.4.99.18	P61806	Dad1	1	0.115180081	0.180992851	0.065812769
S-adenosylmethionine synthase isoform type-2	EC 2.5.1.6	P18298	Mat2a	1	0.037481668	0.159209384	0.121727716
Serine/threonine-protein kinase VRK1 isoform a	EC 2.7.11.1	Q80X41	Vrk1	1	0.091142922	0.205754141	0.114611219
Microsomal glutathione S-transferase 1	EC 2.5.1.18	P08011	Mgst1	2	0.234880268	0.203988231	−0.030892038
Glutathione S-transferase P	EC 2.5.1.18	P04906	Gstp1	3	−0.168833409	−0.247907738	−0.079074329
Phosphatidylinositol 5-phosphate 4-kinase type-2 alpha	EC 2.7.1.149	Q9R0I8	Pip4k2a	3	−0.143480491	−0.17807905	−0.034598559
Protein-tyrosine kinase 2-beta	EC 2.7.10.2	P70600	Ptk2b	3	−0.084516425	−0.157783059	−0.073266634
Serine/threonine-protein kinase OSR1	EC 2.7.11.1	Q6P9R2	Oxsr1	3	−0.118920844	−0.165596042	−0.046675198
Serine/threonine-protein kinase 17B	EC 2.7.11.1	Q91XS8	Stk17b	3	−0.135846465	−0.179465752	−0.043619286
Serine/threonine-protein kinase 4	EC 2.7.11.1	Q9JI11	Stk4	3	−0.050646666	−0.090317152	−0.039670486
5'-AMP-activated protein kinase catalytic subunit alpha-1	EC 2.7.11.1	P54645	Prkaa1	3	−0.101888187	−0.111546268	−0.009658081
Protein kinase C delta type	EC 2.7.11.13	P09215	Prkcd	3	−0.120430329	−0.183010542	−0.062580213
3-ketoacyl-CoA thiolase, mitochondrial	EC 2.3.1.16	P13437	Acaa2	4	0.082421511	−0.127697255	−0.210118766
Lysophosphatidylcholine acyltransferase 2	EC 2.3.1.23	Q8BYI6	Lpcat2	4	−0.181813638	−0.511139267	−0.329325629
1,4-alpha-glucan-branching enzyme	EC 2.4.1.18	Q9D6Y9	Gbe1	4	−0.042286018	−0.148930328	−0.10664431
Uridine phosphorylase 1	EC 2.4.2.3	P52624	Upp1	4	−0.083242608	−0.392550565	−0.309307957
Hypoxanthine-guanine phosphoribosyltransferase	EC 2.4.2.8	Q64531	Hprt1	4	−0.024144311	−0.083055294	−0.058910983
Hexokinase-3	EC 2.7.1.1	P27926	Hk3	4	−0.016624745	−0.117983675	−0.10135893
6-phosphofructokinase, liver type	EC 2.7.1.11	P12382	Pfkl	4	−0.071999766	−0.176198903	−0.104199137
N-acetylgalactosamine kinase	EC 2.7.1.157	Q5XIG6	Galk2	4	−0.038659611	−0.179037398	−0.140377787
Pyruvate kinase PKM	EC 2.7.1.40	P11980	Pkm	4	−0.055693459	−0.118552564	−0.062859105
Tyrosine-protein kinase Lyn isoform A	EC 2.7.10.2	Q07014	Lyn	4	−0.020298629	−0.15028629	−0.129987661
Tyrosine-protein kinase Fgr	EC 2.7.10.2	Q6P6U0	Fgr	4	−0.046943698	−0.136581547	−0.089637849
Ribosomal protein S6 kinase alpha-3	EC 2.7.11.1	P18654	Rps6ka3	4	−0.073325148	−0.182621537	−0.109296389
Pyruvate dehydrogenase kinase, isozyme 3	EC 2.7.11.2	Q922H2	Pdk3	4	−0.04791905	−0.361413111	−0.313494062
3-mercaptopyruvate sulfurtransferase	EC 2.8.1.2	P97532	Mpst	4	−0.083073726	−0.202096667	−0.119022941
Interferon-induced, double-stranded RNA-activated protein kinase	EC 2.7.11.1	Q63184	Eif2ak2	5	−0.360888899	−0.108859607	0.252029292
**HYDROLASES**							
Palmitoyl-protein thioesterase 1 precursor	EC 3.1.2.22	P45479	Ppt1	1	0.24879684	0.347257042	0.098460201
Eukaryotic translation initiation factor 3 subunit F	EC 3.4.19.12	Q9DCH4	Eif3f	1	0.108275952	0.155827424	0.047551472
Caspase-1	EC 3.4.22.36	P43527	Casp1	1	0.103221535	0.121577846	0.01835631
Cathepsin D	EC 3.4.23.5	P24268	Ctsd	1	0.133927025	0.311194503	0.177267478
N-acylethanolamine-hydrolyzing acid amidase precursor	EC 3.5.1.-	Q5KTC7	Naaa	1	0.085215327	0.277437413	0.192222086
DNA replication licensing factor MCM2	EC 3.6.4.12	P97310	Mcm2	1	0.056502266	0.187656256	0.13115399
DNA replication licensing factor MCM3	EC 3.6.4.12	P25206	Mcm3	1	0.150264288	0.218667178	0.06840289
DNA replication licensing factor MCM4	EC 3.6.4.12	P49717	Mcm4	1	0.126913574	0.250998827	0.124085253
DNA replication licensing factor MCM5	EC 3.6.4.12	P49718	Mcm5	1	0.105563621	0.199463388	0.093899767
DNA replication licensing factor MCM6	EC 3.6.4.12	P97311	Mcm6	1	0.076101678	0.193375561	0.117273883
DNA replication licensing factor MCM7	EC 3.6.4.12	Q61881	Mcm7	1	0.064784112	0.346338741	0.281554628
Chromodomain-helicase-DNA-binding protein 4	EC 3.6.4.12	Q6PDQ2	Chd4	1	0.032541132	0.099751668	0.067210535
Eukaryotic initiation factor 4A-I isoform 1	EC 3.6.4.13	P60843	Eif4a1	1	0.128043156	0.286460547	0.158417392
Probable ATP-dependent RNA helicase DDX5	EC 3.6.4.13	Q61656	Ddx5	1	0.110607888	0.189602631	0.078994744
Myeloblastin	EC 3.4.21.76	Q61096	Prtn3	2	0.478989991	−0.295825849	−0.77481584
Neutral cholesterol ester hydrolase 1	EC 3.1.1.-	B2GV54	Nceh1	3	−0.170985165	−0.281472967	−0.110487802
Eosinophil cationic protein precursor	EC 3.1.27.-	P70709	Ear11	3	−1.094986988	−1.472034709	−0.377047722
Protein phosphatase 1F	EC 3.1.3.16	Q9WVR7	Ppm1f	3	−0.15635207	−0.217872783	−0.061520713
Inositol monophosphatase 1	EC 3.1.3.25	P97697	Impa1	3	−0.13409359	−0.123614044	0.010479545
Mast cell carboxypeptidase A	EC 3.4.17.1	P21961	Cpa3	3	−2.502754553	−2.166354088	0.336400465
Mast cell protease 1 preproprotein	EC 3.4.21.39	P09650	Mcpt1l1	3	−2.243417189	−1.066062508	1.177354681
Chymase precursor	EC 3.4.21.39	P50339	Cma1	3	−1.929537438	−0.974073498	0.95546394
Protein-arginine deiminase type-4	EC 3.5.3.15	O88807	Padi4	3	−0.072190624	−0.125052572	−0.052861949
Ectonucleoside triphosphate diphosphohydrolase 1	EC 3.6.1.5	P97687	Entpd1	3	−0.486430431	−0.642852938	−0.156422508
Putative helicase MOV-10 isoform b	EC 3.6.4.13	P23249	Mov10	3	−0.562804813	−0.622111451	−0.059306638
Acyl-protein thioesterase 2	EC 3.1.2.-	Q9QYL8	Lypla2	4	−0.101689793	−0.218873152	−0.117183359
Receptor-type tyrosine-protein phosphatase C isoform 4 precursor	EC 3.1.3.48	P04157	Ptprc	4	−0.097829627	−0.215167164	−0.117337537
Phosphatidylinositol 3,4,5-trisphosphate 5-phosphatase 1	EC 3.1.3.86	P97573	Inpp5d	4	−0.027078682	−0.067062757	−0.039984075
Beta-hexosaminidase subunit beta precursor	EC 3.2.1.52	Q6AXR4	Hexb	4	−0.031166072	−0.116565045	−0.085398973
Leukotriene A-4 hydrolase	EC 3.3.2.6	P24527	Lta4h	4	0.196955814	−0.205664033	−0.402619848
Aminopeptidase N precursor	EC 3.4.11.2	P15684	Anpep	4	−0.058052056	−0.136747257	−0.078695201
Leucyl-cystinyl aminopeptidase isoform 1	EC 3.4.11.3	P97629	Lnpep	4	0.024568668	−0.161756158	−0.186324826
Dipeptidyl peptidase 1 precursor	EC 3.4.14.1	P80067	Ctsc	4	−0.030607236	−0.175121607	−0.144514371
Gamma-glutamyltransferase 5	EC 3.4.19.13	Q9QWE9	Ggt5	4	−0.024176962	−0.279898505	−0.255721543
Neutrophil elastase precursor	EC 3.4.21.37	Q3UP87	Elane	4	0.216634577	−0.233671599	−0.450306176
Signal peptidase complex catalytic subunit SEC11A	EC 3.4.21.89	P42667	Sec11a	4	−0.053486912	−0.112319915	−0.058833002
Diphosphoinositol polyphosphate phosphohydrolase 2	EC 3.6.1.52	Q8R2U6	Nudt4	4	−0.159124629	−0.324161988	−0.165037359
Vesicle-fusing ATPase	EC 3.6.4.6	P18708	Nsf	4	−0.034479238	−0.137419351	−0.102940112
Dynamin-2	EC 3.6.5.5	P39052	Dnm2	4	−0.002743949	−0.079469533	−0.076725584
**LYASES**							
40S ribosomal protein S3	EC 4.2.99.18	P62908	Rps3	1	0.196114129	0.350698421	0.154584292
DNA-(apurinic or apyrimidinic site) lyase	EC 4.2.99.18	P43138	Apex1	3	−0.085613485	−0.139340232	−0.053726747
Argininosuccinate lyase	EC 4.3.2.1	P20673	Asl	4	−0.045119034	−0.12824703	−0.083127997
**ISOMERASES**							
Peptidyl-prolyl cis-trans isomerase B	EC 5.2.1.8	P24368	Ppib	1	0.050687673	0.110150995	0.059463322
Peptidyl-prolyl cis-trans isomerase FKBP3	EC 5.2.1.8	Q62446	Fkbp3	1	0.099918153	0.244218789	0.144300636
Peptidyl-prolyl cis-trans isomerase D	EC 5.2.1.8	Q6DGG0	Ppid	1	0.050889252	0.096319227	0.045429975
Protein disulfide-isomerase	EC 5.3.4.1	P04785	P4hb	1	0.070606559	0.218578766	0.147972206
Protein disulfide-isomerase A3	EC 5.3.4.1	P11598	Pdia3	1	0.085046824	0.153754172	0.068707347
Protein disulfide-isomerase A4	EC 5.3.4.1	P38659	Pdia4	1	0.072096175	0.155111366	0.083015192
Protein disulfide-isomerase A6	EC 5.3.4.1	Q63081	Pdia6	1	0.108211498	0.208593967	0.100382469
Peptidyl-prolyl cis-trans isomerase F, mitochondrial precursor	EC 5.2.1.8	P29117	Ppif	2	0.581369784	0.210543386	−0.370826398
Macrophage migration inhibitory factor	EC 5.3.2.1	P30904	Mif	2	0.461822345	−0.148221794	−0.610044139
Ribulose-phosphate 3-epimerase	EC 5.1.3.1	Q8VEE0	Rpe	4	−0.174094827	−0.354098654	−0.180003827
NAD(P)H-hydrate epimerase	EC 5.1.99.6	B0BNM1	Apoa1bp	4	−0.03634563	−0.110532371	−0.074186741
**LIGASES**							
Aspartate–tRNA ligase, cytoplasmic	EC 6.1.1.12	P15178	Dars	1	0.047262581	0.090565223	0.043302641
Leucine–tRNA ligase, cytoplasmic	EC 6.1.1.4	Q8BMJ2	Lars	1	0.107642729	0.217467451	0.109824722
E3 ubiquitin-protein ligase NEDD4	EC 6.3.2.-	Q62940	Nedd4	1	0.086895543	0.239777618	0.152882074
Ubiquitin-conjugating enzyme E2 L3	EC 6.3.2.19	P68037	Gm10705	1	0.02016907	0.091055143	0.070886073
Asparagine synthetase [glutamine-hydrolyzing]	EC 6.3.5.4	P49088	Asns	1	0.166112474	0.242415172	0.076302699
Glycine–tRNA ligase	EC 6.1.1.14	Q5I0G4	Gars	2	0.119496174	0.008715768	−0.110780406
Nicotinate phosphoribosyltransferase	EC 6.3.4.21	Q6XQN1	Naprt1	4	0.091298572	−0.136451197	−0.227749769
Methionine–tRNA ligase, cytoplasmic isoform 2	EC 6.1.1.10	Q68FL6	Mars	5	−0.056877927	0.129932982	0.186810909

### Major functional classes of the neutrophil proteome regulated by IR

**Figure 4:**
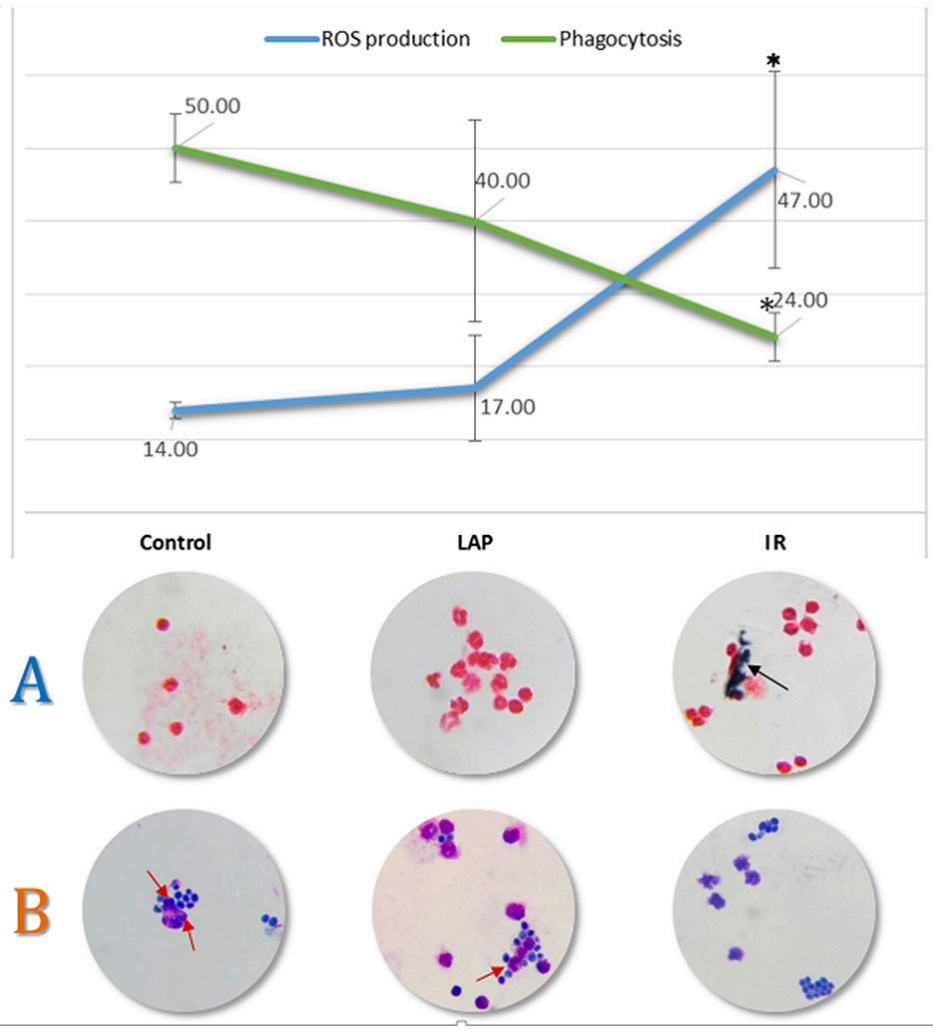
Evaluation of ROS production and phagocytosis. The line graph shows the results of neutrophil function assays obtained by optical microscopy analysis—Phagocytosis in green and ROS production in blue. The asterisk represents significant differences (*p* < 0.05) when compared to control or to LAP groups. Cell counts were normalized to one hundred cells. Typical cells are shown below their respective condition in the graph for ROS evaluation **(A)** where the black arrow points to formazan crystals and phagocytosis **(B)** of *Saccharomyces cerevisiae* pointed by red arrows.

For the functional classification of the significantly regulated identified proteins, KEGG and Wiki pathways analyses were done and the enriched pathways are listed in Table 2. Most of the enriched pathways found were immune-related indicating the effect of intestinal ischemia and reperfusion on the neutrophil function. Phagocytosis was found significantly down regulated in IR as shown in Table 2, Figure 4.

**Table 2:** Major functional classification.

**Protein ID**	**Gene symbol**	**Protein name**	**Cluster**	**LAP vs. Ctrl Log2 ratio**	**IR vs. Ctrl Log2 ratio**	**IR vs. LAP Log2 ratio**
**KEGG: Spliceosome** ***(rawP** = **1.13e-05; adjP** = **5.09e-05)***						
P63017	Hspa8	Heat shock 70kDa protein 8	1	0.045693617	0.106185806	0.06049219
O35900	Lsm2	LSM2 homolog, U6 small nuclear RNA associated (S. cerevisiae)	1	0.067225314	0.155666556	0.088441241
O35326	Srsf5	Serine/arginine-rich splicing factor 5	1	0.144719584	0.239436591	0.094717008
Q8VE97	Srsf4	Serine/arginine-rich splicing factor 4	1	0.048158793	0.118255729	0.070096936
Q61656	Ddx5	DEAD (Asp-Glu-Ala-Asp) box helicase 5	1	0.110607888	0.189602631	0.078994744
P60335	Pcbp1	Poly(rC) binding protein 1	1	0.104001931	0.13900654	0.035004608
**KEGG: antigen processing and presentation** ***(rawP** = **2.26e-05; adjP** = **8.72e-05)***						
P63017	Hspa8	Heat shock 70kDa protein 8	1	0.045693617	0.106185806	0.06049219
P06761	Hspa5	Heat shock protein 5	1	0.10315848	0.165189029	0.06203055
P11499	Hsp90ab1	Heat shock protein 90 alpha (cytosolic), class B member 1	1	0.062540962	0.274247984	0.211707022
P82995	Hsp90aa1	Heat shock protein 90, alpha (cytosolic), class A member 1	1	0.034297768	0.130094309	0.095796541
P11598	Pdia3	Protein disulfide isomerase family A, member 3	1	0.085046824	0.153754172	0.068707347
**KEGG: NOD-like receptor signaling pathway** ***(rawP** = **4.45e-05; adjP** = **0.0002)***						
P43527	Casp1	Caspase 1	1	0.103221535	0.121577846	0.01835631
P11499	Hsp90ab1	Heat shock protein 90 alpha (cytosolic), class B member 1	1	0.062540962	0.274247984	0.211707022
P82995	Hsp90aa1	Heat shock protein 90, alpha (cytosolic), class A member 1	1	0.034297768	0.130094309	0.095796541
Q66HD0	Hsp90b1	Heat shock protein 90, beta, member 1	1	0.12678686	0.188938948	0.062152088
**Wikipathways: TNF-alpha NF-kB signaling pathway** ***(rawP** = **9.40e-07; adjP** = **2.98e-06)***						
P50878	Rpl4	Ribosomal protein L4	1	0.143724339	0.304591572	0.160867233
P49718	Mcm5	Minichromosome maintenance complex component 5	1	0.105563621	0.199463388	0.093899767
P11499	Hsp90ab1	Heat shock protein 90 alpha (cytosolic), class B member 1	1	0.062540962	0.274247984	0.211707022
P68040	Gnb2l1	Guanine nucleotide binding protein (G protein), beta polypeptide 2 like 1	1	0.198001407	0.364800338	0.166798931
P82995	Hsp90aa1	Heat shock protein 90, alpha (cytosolic), class A member 1	1	0.034297768	0.130094309	0.095796541
P21533	Rpl6	Ribosomal protein L6	1	0.100057017	0.30453836	0.204481343
Q61881	Mcm7	Minichromosome maintenance complex component 7	1	0.064784112	0.346338741	0.281554628
P62281	Rps11	Ribosomal protein S11	1	0.180726804	0.335684506	0.154957702
**Wikipathways: MAPK signaling pathway** ***(rawP** = **0.0033; adjP** = **0.0074)***						
P63017	Hspa8	Heat shock 70kDa protein 8	1	0.045693617	0.106185806	0.06049219
P43527	Casp1	Caspase 1	1	0.103221535	0.121577846	0.01835631
P06761	Hspa5	Heat shock protein 5	1	0.10315848	0.165189029	0.06203055
P13668	Stmn1	Stathmin 1	1	0.10969688	0.252735553	0.143038673
**Wikipathways: IL-2 signaling pathway** ***(rawP** = **0.0047; adjP** = **0.0089)***						
P68040	Gnb2l1	Guanine nucleotide binding protein (G protein), beta polypeptide 2 like 1	1	0.198001407	0.364800338	0.166798931
P82995	Hsp90aa1	Heat shock protein 90, alpha (cytosolic), class A member 1	1	0.034297768	0.130094309	0.095796541
P52631	Stat3	Signal transducer and activator of transcription 3 (acute-phase response factor)	1	0.040131681	0.155755431	0.115623749
**KEGG: Fc gamma R-mediated phagocytosis** ***(rawP** = **2.74e-06; adjP** = **6.17e-05)***						
Q8BH43	Wasf2	WAS protein family, member 2	4	−0.046564123	−0.154032936	−0.107468813
P04157	Ptprc	Protein tyrosine phosphatase, receptor type, C	4	−0.097829627	−0.215167164	−0.117337537
Q07014	Lyn	V-yes-1 Yamaguchi sarcoma viral related oncogene homolog	4	−0.020298629	−0.15028629	−0.129987661
P39052	Dnm2	Dynamin 2	4	−0.002743949	−0.079469533	−0.076725584
P97573	Inpp5d	Inositol polyphosphate-5-phosphatase D	4	−0.027078682	−0.067062757	−0.039984075
**KEGG: regulation of actin cytoskeleton** ***(rawP** = **0.0001; adjP** = **0.0005)***						
Q8BH43	Wasf2	WAS protein family, member 2	4	−0.046564123	−0.154032936	−0.107468813
P11835	Itgb2	Integrin, beta 2	4	0.039783401	−0.134544799	−0.1743282
P27601	Gna13	Guanine nucleotide binding protein (G protein), alpha 13	4	0.027347849	−0.171688468	−0.199036317
Q5SQX6	Cyfip2	Cytoplasmic FMR1 interacting protein 2	4	−0.077078437	−0.151342467	−0.07426403
Q62812	Myh9	Myosin, heavy chain 9, non-muscle	4	−0.032033077	−0.082815246	−0.050782169
**KEGG: Chemokine signaling pathway** ***(rawP** = **7.05e-05; adjP** = **0.0005)***						
P04897	Gnai2	Guanine nucleotide binding protein (G protein), alpha inhibiting activity polypeptide 2	4	0.013197352	−0.127231852	−0.140429204
P62994	Grb2	Growth factor receptor bound protein 2	4	−0.034963374	−0.080293598	−0.045330224
Q07014	Lyn	V-yes-1 Yamaguchi sarcoma viral related oncogene homolog	4	−0.020298629	−0.15028629	−0.129987661
Q6P6U0	Fgr	Gardner-Rasheed feline sarcoma viral (v-fgr) oncogene homolog	4	−0.046943698	−0.136581547	−0.089637849
Q9CXP8	Gng10	Guanine nucleotide binding protein (G protein), gamma 10	4	0.027942431	−0.231202992	−0.259145422
**Biological process: leukotriene biosynthetic process** ***(rawP** = **3.87e-06; adjP** = **0.0010)***						
Q02759	Alox15	Arachidonate 15-lipoxygenase	4	−0.591830552	−1.210220167	−0.618389614
P20291	Alox5ap	Arachidonate 5-lipoxygenase activating protein	4	−0.023100206	−0.204507854	−0.181407647
P24527	Lta4h	Leukotriene A4 hydrolase	4	0.196955814	−0.205664033	−0.402619848
Q9QWE9	Ggt5	Gamma-glutamyltransferase 5	4	−0.024176962	−0.279898505	−0.255721543
**Biological process: response to bacterium** ***(Rawp** = **8.09e-05; Adjp** = **0.0015)***						
P51437	Camp	Cathelicidin antimicrobial peptide	5	−0.158024975	−0.007959577	0.150065397
P42225	Stat1	Signal transducer and activator of transcription 1	5	−0.13682191	0.04853719	0.1853591
Q63184	Eif2ak2	Eukaryotic translation initiation factor 2-alpha kinase 2	5	−0.360888899	−0.108859607	0.252029292
**Biological process: response to reactive oxygen species** ***(rawP** = **1.90e-05; adjP** = **0.0016)***						
P04906	Gstp1	Glutathione S-transferase pi 1	3	−0.168833409	−0.247907738	−0.079074329
P70600	Ptk2b	PTK2B protein tyrosine kinase 2 beta	3	−0.084516425	−0.157783059	−0.073266634
P09215	Prkcd	Protein kinase C, delta	3	−0.120430329	−0.183010542	−0.062580213
P43138	Apex1	APEX nuclease (multifunctional DNA repair enzyme) 1	3	−0.085613485	−0.139340232	−0.053726747
P54645	Prkaa1	Protein kinase, AMP-activated, alpha 1 catalytic subunit	3	−0.101888187	−0.111546268	−0.009658081
P11672	Lcn2	Lipocalin 2	3	−0.538858464	−0.554555684	−0.01569722
P04041	Gpx1	Glutathione peroxidase 1	3	−0.131894129	−0.236200267	−0.104306138

*rawP, p-value from hypergeometric test*.

*adjP, p-value adjusted by the multiple test adjustment*.

### Verification of ROS production and phagocytosis

To validate such findings we performed a ROS production and phagocytosis assays by incubating neutrophils from the three groups with *Saccharomyces cerevisiae* yeast cells. Phagocytic activity was significantly decreased in IR group (*p*-value < 0.05) compared to control and LAP (Figure 4). Only about 23.90% of cells phagocytosed in IR group while control and LAP presented 50 and 40.7% phagocytosis rates respectively.

## Discussion

Speculating about correlations between cluster profiles and physiological conditions, cluster 1 suggests possible markers that would correlate to the injury severity, since protein abundances progressively increase with the severity of the surgical event. Clusters 2 and 5 suggest proteins that change the sense of regulation when the condition changes from mild to more intense surgical procedure and, in similar ways, cluster 3 detected proteins that respond similarly to any intensity of trauma, and proteins regulated only by intestinal ischemia and reperfusion were clustered in 4.

The five clusters where our quantified proteins were grouped did not enrich for specific cellular localization. According to Supplementary Figure S1A, most of the significantly regulated proteins from clusters 1, 3, and 4 were annotated to cytoplasm, extracellular, and nucleus. Cytosol and organelle lumen had proteins from cluster 1 whereas ribosome proteins were exclusively annotated to cluster 1. Mostly membrane proteins were grouped in cluster 4 that is down regulated in IR as compared to the control and laparotomy groups.

Proteins related to metabolic process from cluster 1 and 4 showed enrichment (Supplementary Figure S1B) whereas, the highest number of proteins related to metabolic processes belong to cluster 1 which is continuously up regulated in laparotomy and IR as compared to the control group. Gene Ontology (GO) terms distribution for the molecular functions (Supplementary Figure S1C) of all the regulated proteins from clusters 1, 3, and 4 showed annotation to catalytic activity, binding proteins whereas cluster 1 represented a high amount of protein enrichment for RNA binding and structural molecule activity. Proteins from cluster 1 and 4 showed highest diversity in molecular functions among the clusters.

After identifying a large number of proteins, the data were analyzed in Uniprot to predict the enzyme activity for the identified neutrophil proteome. It has been clear from the literature that different enzymes play an important role in neutrophils. The enzyme activities encountered varies for each cluster (Figure 3B).

### Predicted enzyme activity for cluster 1 proteins

These enzymes are found progressively up regulated in neutrophils from the control to the laparotomy to the ischemia/reperfusion group suggesting that the intensity of regulation may be proportional to the severity of the inflammatory process.

#### Oxidoreductases

The quantitative analysis revealed significantly up regulated oxidoreductases in cluster 1, among which we found superoxide dismutase (SOD). Normally during inflammation, SOD regulates ROS concentration and reactive nitrogen species however high levels of extracellular SOD activity resulted in reduced innate immune response of neutrophils (Break et al., 2012). Increased level of DHFR was observed in our quantitative analysis whereas in peripheral blood leucocytes, mainly neutrophils, of cancer patients' higher expression of DHFR has been associated with leukocytosis (Iqbal et al., 2000). Other significantly up regulated oxidoreductases of cluster 1 include Prostagladin reductase 1 (PtGR-1), and fatty acyl-CoA reductase 1 (Table 1). PtGR-1 is a nitroalkene reductase and its overexpression in HEK293T cells promoted inactivation of nitroalkane, inhibition of hemeoxygenase HO-1 and finally abrogated tissue protection (Vitturi et al., 2013). It has been shown that inducing HO-1 prior to IRI results in a significant decrease of intestinal tissue injury (Wasserberg et al., 2007).

#### Transferases

N-myristoyltransferase (NMT1) catalyzes protein myristoylation process that is essential for leukocyte growth and development, found elevated in activated neutrophils along with increase in lifespan (Kumar et al., 2011). The majority of significantly regulated transferases are oligosaccharyltransferases involved in *N*-Linked protein glycosylation. Since pro-inflammatory stimuli modify glycan profiles on cell surfaces, the overexpression of such enzymes can lead to an increased adhesion of activated neutrophils to endothelial cells (Sriramarao et al., 1993). S-adenosylmethionine (SAM) acts as a donor for methyltransferases that is involved in methylation of DNA, RNA, proteins, and lipids. SAM has important implications in antigen-induced immune responses (Wu et al., 2005).

#### Hydrolases

Most of the regulated hydrolases are annotated to DNA helicases and one RNA helicase. Other statistically regulated hydrolases, relevant to the inflammation were Caspase-1, involved in the inflamasome pathway, and Cathepsin D. Increased activity of cathepsin D in myocardial ischemic neutrophils was found to be associated with increased ROS production (Miriyala et al., 2013). Both the Caspase-1 and Cathepsin D were significantly found up regulated in this study showing high inflammasome pathway activation and ROS production.

### Predicted enzyme activity for cluster 4 proteins

Another group of transferases, oxidoreductases, and hydrolases present predominant regulation in neutrophils from intestinal ischemia/reperfusion in cluster 4 as shown in Figure 3B.

#### Oxidoreductases

There are some important oxidoreductases regulated in this cluster. Arachidonate 15-lipoxygenase was found significantly down regulated. It has been known that arachidonate 12/15-lipoxygenases (12/15-LOX) forms hydroperoxy eicosatetraenoic acids (HPETEs) from arachidonic acid that subsequently produces eicosanoids. Leukocytes highly expresses 15-LOX but little is known about its role in neutrophils (Nadel et al., 1991). Xanthine oxidase (Xdh) and thioredoxin reductase 1 (TXNRD1) are cellular defense enzymes against oxidative stress. Significantly down regulation of these antioxidants after IR can be an important step in modulating the neutrophil response to oxidative stress (Vorbach et al., 2003; Biterova et al., 2005). The most extensively studied primary granule enzyme of neutrophils is myeloperoxidase (MPO), found significantly regulated in cluster 4 by our quantitative analysis (Table 1). Recently an increase in MPO along with ICAM-1 and P-selectin expressions in neutrophils has been found in a model of small intestinal ischemia in rats (Gan et al., 2015).

#### Transferases

Most down regulated transferases have function in phospholipid metabolism, carbohydrate degradation (glycolysis) and nucleotide metabolism. The tyrosine-protein kinases Lyn/Fgr belong to the Src family of kinases that negatively regulate integrin-signaling pathway. Neutrophils deficient of these kinases had enhanced respiratory burst, secondary granule release, and a hyper-adhesive phenotype due to reduced mobilization of SHP-1 (Pereira and Lowell, 2003). Many metabolic enzymes including hexokinase, phosphofructokinase and pyruvate kinase were found significantly down regulated in cluster 4 in this study, changes that have also been previously observed during neutrophil activation (Huang et al., 2002). Pyruvate kinase down regulation can lead to partial inhibition of glycolysis at the last step and enhance the synthesis of lipids and nucleic acids, however neutrophils deficient in PK also showed immunodeficiency (Burge et al., 1976). At the first step of glycolysis, Hexokinase (HK3) has 70–80% activity in granulocytes while its down regulation impairs neutrophil differentiation (Federzoni et al., 2012). Although significantly regulated, the transposition of these results to functional inferences needs validation due to the presence of multiple proteoforms of such enzymes.

#### Hydrolases

Some of the important hydrolases were found significantly down regulated like P97573, Phosphatidylinositol 3,4,5-trisphosphate 5-phosphatase (SHIP). Neutrophil with loss of SHIP showed defective cell migration, loss of polarity upon cell adhesion and increased adhesion due to Akt activation and higher PtdIns(3,4,5)P3 (Mondal et al., 2012). Leukotrienes (LTs) are implicated in a wide variety of inflammatory disorders and are produced in arachidonic acid (Kutmon et al., 2015) cascade in immune cells. Two bifunctional hydrolases of AA cascade were found regulated in cluster 4. One is leukotriene A-4 hydrolase (LTA4H) involved in leukotriene B4 biosynthesis from LTB_4_ (Liu and Yokomizo, 2015) and in aminopeptidase activity by breakdown and clearance of Proline-Glycine-Proline (PGP), a neutrophil chemoattractant (Snelgrove et al., 2010). The other bifunctional hydrolase found down regulated, Gamma-glutamyltransferase 5 (GGT5), participates in glutathione metabolism and in leukotriene D4 biosynthesis from LTC_4_ and down regulation of these enzymes can lead to accumulation of leukotriene products (Liu and Yokomizo, 2015) along with PGP and affect neutrophil biology with influx of neutrophils into the tissue and air spaces (Paige et al., 2014). Another interesting hydrolase is Dynamin-2\Dynamin GTPase (DNM), which is involved in microtubules production, binding and hydrolyzation of GTP. Recently, a study showed that inhibition of dynamin impaired the membrane fusion/fission events and resulted in production of highly adhesive cellular secretory protrusions called cytonemes that help neutrophils in long-range contacts with other cells or bacteria after adhesion. Down regulation of dynamin can be an important step in cytoneme production leading to increase in adhesion (Galkina et al., 2005).

An overall evaluation of the enzymes regulated in cluster 1 suggests that neutrophils would progressively increase the ROS production and self-protection against ROS (like SOD, DHFR, Cathepsin-D), decrease tissue protection e.g. PtGR1, increase neutrophil lifespan such as NMT1 and promote adhesion (oligosaccharyl transferases). Such progression appears to be proportional to the injury severity. The regulated enzymes in cluster 4 also suggest an intense effect of IR on the oxidative stress mechanism (including XO, TXNRD1, MPO, Lyn, Fgr, and GGt5) and leukotriene metabolism (as LTA4H and GGT5).

To validate the results regarding ROS production, we performed an NBT test that allows the visualization of blue formazan crystals inside cells that produced ROS. We compared the formazan crystals formation in control, LAP and IR surgical groups. Yellow and water-soluble NBT is reduced to blue formazan crystals by ROS produced by activated neutrophils (Baehner and Nathan, 1968). The exposure of neutrophils to intestinal IR induced a significantly higher (*p* < 0.05) amount of formazan (Figure 4), used as a marker of NADPH oxidase activity: 46.5% of the cells contained extensive formazan formation while LAP and control showed 16.9% and 14.4% cells respectively, probably due to baseline production. These results support the hypothesis that neutrophils contribute to the ischemic oxidative stress as shown in (Jaeschke et al., 1992; Arumugam et al., 2004) and may be related to the regulation of antioxidant molecules after IR found in cluster 4, as well as the regulation of SOD, DHFR, and Cathepsin-D found in cluster 1.

### Major functional classes of neutrophil proteome

For the functional classification of the significantly regulated identified proteins, KEGG pathways, and Wikipathways databases were used as a reference knowledge base to understand various signaling mechanisms and pathways (Zhang and Wiemann, 2009; Kutmon et al., 2015). Differentially regulated proteins were mapped to the *Rattus norvegicus* genome as reference set for enrichment analysis using the online analysis WebGestalt (Wang et al., 2013). Most of the enriched pathways are immune-related indicating the effect of intestinal ischemia and reperfusion on the neutrophil function.

Five proteins from cluster 1 were found to be involved in antigen processing and presentation. They are Hspa8 (heat shock 70kDa protein 8), Hspa5 (heat shock protein 5), Hsp90aa1/Hsp90ab1 [heat shock protein 90, alpha (cytosolic), class A member 1/class B member 1] and Pdia3 (protein disulfide isomerase family A, member 3). Antigen processing and presentation is a well-known phenomenon performed by antigen-presenting cells, including dendritic cells, B cells, macrophages, and thymic epithelial cells, signaling to antigen-specific T cells in order to generate effective adaptive immune responses (Roche and Furuta, 2015). It has been found recently that mouse neutrophils express MHC class II and co-stimulatory molecules like CD80 and CD86, and can prime antigen-specific T cells in an MHC class II-dependent manner (Abi Abdallah et al., 2011). Interestingly the identification of up regulation of the identified proteins in our data set suggests that intestinal ischemia and reperfusion increases antigen processing and presentation process in neutrophils.

TNF-alpha/NF-kB signaling pathway was significantly enriched with eight proteins from cluster 1. TNF-alpha is a multifunctional proinflammatory cytokine, which can induce a wide range of apoptosis and cell survival genes as well as inflammation and immunity-related genes by the NF-kB signaling pathway (Barnes and Karin, 1997; Blackwell and Christman, 1997). It has been shown that up-regulation of TNF-alpha/NF-kB signaling pathway after ischemia and reperfusion induces inflammation and tissue injury by the activated neutrophils (Kin et al., 2006). The identification of up-regulation of eight proteins in this pathway expands the previous results that showed up-regulation of this pathway in activated neutrophils after intestinal ischemia and reperfusion causing more injury to the bystander tissues by ROS production (Tang et al., 2011).

Three proteins with significant up-regulation from cluster 1 were grouped in IL-2 signaling pathway with significant enrichment. They include Gnb2l1 [Guanine nucleotide binding protein (G protein), beta polypeptide 2 like 1], Hsp90aa1 [Heat shock protein 90, alpha (cytosolic), class A member 1], and Stat3 (Signal transducer and activator of transcription 3). Jablons et al. (1990) demonstrated that *in vivo* administration of IL-2 in humans with advanced cancers suppresses FcγR expression (CD16) and chemotaxis in neutrophils whereas another study had opposite results and was performed *in vitro* to check the direct effect of IL-2 on the neutrophils functions (Girard et al., 1996). Our results are in accordance with the *in vivo* analysis as our results show up-regulated IL-2 signaling pathway proteins concomitantly with a down regulation of FcγR signaling proteins after IR. Fc gamma R-mediated phagocytosis down regulation was found in cluster 4 (Table 2) and is further discussed below.

Here Fc gamma R mediated phagocytosis, regulation of actin cytoskeleton and chemokine signaling pathways were found down regulated in IR, as described by the cluster 4 profile. All the down regulated proteins in these pathways could lead to dramatically slower cell migration, improper polarization, transendothelial migration (TEM), and phagocytosis. Studies have reported that WASF requirement for initial spike in actin polymerization correlates with directional sensing. Zhang et al. (2006) observed reduced adhesion followed by reduced migration toward fMLP along with reduced TEM in WASF-deficient neutrophils associated with defective β2-integrin clustering. In addition, WASF may play multiple roles in chemotaxis (Kumar et al., 2012).

Disruption of SHIP1\INPP5D, a primary inositol phosphatase causes accumulation of [a PtdIns(3,4,5)P3 probe] and F-actin polymerization across the cell membrane in neutrophils and as a result the neutrophils become flattened and display irregular polarization and less cell migration (Nishio et al., 2007).

We observed down regulation (cluster 4) of Leukotriene synthesis pathways represented by important enzymes like 15 lipoxygenase, 5 lipoxygenase activating protein (FLAP), and LTA_4_ hydrolase. Leukotrienes (LTs) are inflammatory mediators causing, for example, phagocyte chemotaxis and increased vascular permeability. LTB_4_ is a potent chemotactic agent produced by almost all types of immune cells, especially by neutrophils, and its overproduction leads to certain pathological conditions such as lung edema (Pace et al., 2004) and inflammatory bowel disease (Singh et al., 2003). Few studies have shown the effect of down regulation of 5-LO however, one study reported an increased neutrophil infiltration and TNF expression within the myocardial infarction area of 5-LO deficient mice. However inhibition of 5-lipoxygenase did not affect IR related injury of the wild type mice (Adamek et al., 2007). We have also found down regulation of important enzymes of LTB4 synthesis pathway in PMNs after intestinal IR. Similarly, platelets could naturally inhibit the LTB4 synthesis in neutrophils through their spontaneous interactions with these cells. The inhibitory factor can be adenosine as identified in ligand-operated interactions of platelets with neutrophils, but the platelet-derived product responsible for down regulation of neutrophil lipid mediators release and generation remains to be identified. This could have a significant impact on the homeostatic process of inflammation (Chabannes et al., 2003).

It is clear that intestinal obstruction (Sagar et al., 1995) and ischemia (Meddah et al., 2001) cause mucosal injury with a subsequent increase of mucosal permeability and bacterial translocation. Therefore, the up regulation of antimicrobial proteins in cluster 5 like CAMP and Lcn2 observed after IR could be related to protection against bacterial infection and modulation of oxidative stress (Chakraborty et al., 2012).

To validate such findings we performed a phagocytosis assay by incubating neutrophils from the three groups with *Saccharomyces cerevisiae* yeast cells. Phagocytic activity was significantly decreased in the IR group (*p*-value < 0.05) compared to control and LAP (Figure 4). Only about 23.90% of cells phagocytosed in IR group while control and LAP presented 50 and 40.7% phagocytosis rates, respectively. The mechanisms for the decreased neutrophils phagocytosis have not been explained yet, but might be related to the down regulation (cluster 4) of SHIP and the five proteins that belong to the Fc gamma R-mediated phagocytosis pathway (Detmers et al., 1987; Ravetch and Kinet, 1991).

As a result of oxidative stress, most of the tissues undergo various anti-oxidative defense mechanisms. In our study, we found various anti-oxidant proteins in neutrophils that have been significantly regulated after IR. It includes glutathione reductase and glutathione S-transferase (GST). GSTs and its isoenzyme activities increase in response to oxidative stress due to lipid peroxidation resulting from superoxide production (Vasieva, 2011). Glutathione S transferase 1 (GSTP1), is a detoxification enzyme and regulator of cell signaling in response to growth factors, hypoxia, stress, and other stimuli in human hepatocellular carcinoma (HCC) (Kou et al., 2013) but its role in neutrophils is not clear. Down regulation of the response to reactive oxygen species was observed in cluster 3 after IR in neutrophil. Their major role in inflammatory and immune responses has long been thought to be phagocytosis and killing of bacteria via the generation of ROS and release of lytic enzymes stored in granules (Root and Cohen, 1981). Especially ROS, since they are potentially toxic essential molecules in the killing of bacteria. PMNs are thought to be exposed to ROS produced by themselves and by other inflammatory cells, and to suffer from resulting damage, such as DNA cleavage, protein modifications, and lipid peroxidation. The ROS-mediated damage to intracellular molecules is considered to be limited by cellular antioxidant enzymes, such as SODs (superoxide dismutase), Glutathione-s-transferase (Hurst et al., 1998) and GPx (glutathione peroxidase) (Arai et al., 1999). Down regulation of Glutathione-s-transferase and peroxidase could limit PMNs role in inflammatory and immune responses such as phagocytosis and killing of bacteria, as confirmed by our functional assays and previously mentioned in the literature (Hattori et al., 2005).

## Concluding remarks

Conclusively, our proteomic approach revealed that intestinal ischemia/reperfusion causes the down regulation of important antioxidants together with the up regulation of enzymes involved in ROS production. This could result in reactive oxygen species accumulation observed by many researchers and confirmed herein. From enzyme classification, cluster based KEGG pathways analysis and phagocytosis assays, we observed the changes in neutrophils motility, phagocytosis, directional migration, and cytoskeletal machinery activation after ischemia and reperfusion. Moreover, regulated pathways and enzymes suggest the influence of IR on the carbohydrate and lipid metabolism and a possible correlation to bacterial translocation, but such findings need further studies.

Collectively, our MS-based quantitative proteomic analysis illustrates the significance of comparative proteomic strategies applied to the neutrophils in different surgical groups, showing results supported by functional assays and consistent with the literature, and lay the basis for further profound studies in neutrophil biology.

## Ethics statement

This study was carried out in accordance with the recommendations of the Ethics committee at the Faculty of Medicine, University of São Paulo. The protocol was approved by the Ethics committee at the Faculty of Medicine, University of São Paulo, registered under protocol number 8186.

## Author contributions

WF and PR: Study idea and design, data analysis, manuscript writing. MT, SA: sample prep, proteomics analyses, manuscript writing. BF: surgical assays. IL, KB, and MC: functional assays, manuscript writing. SS, VS: data analysis, manuscript writing.

### Conflict of interest statement

The authors declare that the research was conducted in absence of any commercial or financial relationships that could be construed as a potential conflict of interest. The handling editor declared a shared affiliation, though no other collaboration, with several of the authors, MT, SA, MC, IL, KB, and WF, at time of review.

## Abbreviations

CAMP: Cathelicidin antimicrobial peptide
DTT: Dithiothreitol
EC number: Enzyme commission number
FASP: Filter aided sample prep
FDR: False discovery rate
fMLP: N-Formyl-Met-Leu-Phe
GFP-PH-Akt: Green fluorescent protein probe for the PH domain of protein kinase B
HCD: higher energy collisional dissociation
HILIC: Hydrophilic interaction chromatography
IR: Ischemia and reperfusion
iTRAQ: Isobaric tag for relative and absolute quantitation
KEGG: Kyoto Encyclopedia of Genes and Genomes
LC-MS/MS: Tandem mass spectrometry liquid chromatography
Lcn2: Lipocalin-2
LTB4: Leukotriene B4
LTQ: Linear trap quadrupole
MOF: multiple organ failure
PCA: Principal Component Analysis
PMNs: polymorphonuclear leukocytes
PtdIns(3, 4, 5)P3, Phosphatidylinositol (3, 4: 5)-trisphosphate
ROS: Reactive oxygen species
SDS: Sodium dodecyl sulfate
SHP-1: SH2-containing tyrosine-specific protein phosphatase
SIRS: systemic inflammatory response syndrome
TEAB: Triethylammonium bicarbonate
WASF: Wiskott-Aldrich syndrome protein
XC and CC: families of chemokines.
