# Supplementary material for: Analysis of the Effect of Intestinal Ischemia and Reperfusion on the Rat Neutrophils Proteome
Source: Front Mol Biosci. 2018 Nov 29;5:89. doi: 10.3389/fmolb.2018.00089 (PMC6281993; doi:10.3389/fmolb.2018.00089)
Supplement: Supplementary file 3 [file Data_Sheet_1.docx]

**SUPPLEMENTARY METHODS**

**EXPERIMENTAL PROCEDURES**

**Materials**

HPLC grade or higher solvents and chemicals were used during this study. Protease Inhibitor Mix and Quant-iT™ Protein Assay Kit were from GE Healthcare Biosciences (Pittsburgh, USA). PhosSTOP was from Roche – Germany. Iodoacetamide was from GE Healthcare (Amersham, UK). DTT and SDS were from GE Healthcare Bio-Sciences (Uppsala, Sweden). Poros Oligo R2 and R3 reversed-phase material were obtained from PerSeptive Biosystems (Framingham, MA). TEAB was from Sigma Aldrich – Switzerland. Trypsin was from Promega (Madison, WI). iTRAQ reagents were purchased from Applied Biosystems (Foster City, CA). Vivacon 30KDa filters were from Sartorius Stedim Biotech (Goettingen, Germany). TFA was from Fluka (St. Louis, MO). TSKGel Amide-80 2 mm, 3μm particle size was from Tosoh Bioscience (Stuttgart, Germany). ReproSil-Pur C18 AQ 3µm material was from Dr. Maisch, (Ammerbuch-Entringen, Germany). Empore C8 extraction disk was obtained from 3 M Bioanalytical Technologies (St. Paul, MN). All the sample preparation procedures were carried out inlow-binding polypropylene microtubes from Sorenson Bioscience (Salt Lake City, UT).

**Experimental design and statistics**

We used three experimental conditions: Control, Laparotomy and Ischemia/reperfusion. For the proteomics analysis, each condition was represented by a group of 10 rats. After surgery, neutrophil isolation and lysis, the cell lysates were randomized and pooled in pairs within each condition, giving five biological replicates representing ten subjects. For the functional assays of ROS production and phagocytosis, nine more rats were used, being three for each condition. A general overview of the experimental design in given in Fig. 1 in the manuscript.

Statistical validation of the protein identification was based on decoy searches and unique peptides. Only proteins presenting FDR ≤ 1% and at least two peptides, being one of them unique, were considered. For the quantitative proteomic analysis, the iTRAQ reporter ion intensities were log-transformed, median normalized and evaluated for multivariate analysis using standard PCA. Clustering of proteins was defined according to their abundance in the best number of clusters, using two validation indices, Xie-Beni index [1] and minimal centroid distance [2]. The Limma test [3] and rank products [4] tests were used to evaluate the significance of the quantitative difference observed for each protein among the conditions. Proteins with q-value less than 0.05 (5% FDR) were considered significantly regulated. Enrichment statistics for GO terms and pathways was based on hypergeometric test and adjusted for multiple testing. The quantitative aspects of functional assays were based on cell counting and the normalized values were subjected to the t-test.

**Surgical procedures and sample collection**

Male Wistar rats showing no inflammatory symptoms, weighing 250–350g were collected from the animal house of the Faculty of Medicine, University of Sao Paulo (FMUSP), São Paulo State, Brazil. The project was approved by the Ethical Committee at FMUSP under protocol No. 8186. To perform proteomic analysis, thirty rats were blindly divided in three groups where each experimental group had 10 rats. These groups included control, sham laparotomy (LAP) (without clamping of the mesenteric artery, but comprehending the same surgical procedures applied to the IR group), ischemia reperfusion (IR) group (subjected to 120min of reperfusion preceded by 45 min of superior mesenteric artery occlusion).

The Surgical procedures were carried out in the Laboratory of Medical Investigation (LIM-62), department of Surgery, FMUSP according to [5]. A small blood sample was first analyzed by hemocytometery and rats with no sign of inflammation (Data not shown) were selected for further surgical procedures and sample collection. For proteomics studies about 10-12 ml of blood were collected directly from heart (right ventricle catheter).

**Neutrophils separation and protein extraction**

Rat neutrophils were isolated from the blood as previously described in [6] with some modifications and 200 μl of lysis buffer for 3x10^6^ neutrophils (2% SDS, 20mM TEAB, 100mM DTT, protease inhibitors) was added for protein extraction [7]. Neutrophil cells were lysed in the lysis buffer by using a 50W tip-sonicator with 40% output, 10 cycles for 15 s each and 1 min of interval to cool down the sample on ice. Following sonication the samples were heated for 10 minutes at 80°C, centrifuged at 10000 rpm and supernatant was transferred to fresh eppendorf tube.

**Sample preparation**

The protein quantification was performed by using Quant-iT™ Protein Assay Kit. Proteins were processed in Vivacon 30kDa spin filter vials as described by Leon et al. 2013 in order to remove SDS from the samples [8]. The samples were then alkylated (as DTT was already used in the lysis buffer) using 50 mM Iodoacetamide (IAA) for 30 min in dark. After centrifugation the filtrate was discardedand proteins were digested using Promega Trypsin in 1:50 (trypsin to proteins ratio) overnight at 37°C. To stop the digestion reaction, formic acid was added to 1% (v/v) and peptides were collected by centrifuging at 15, 000rpm for 10 min. The peptides were desalted as previously described [7]. Prior to lyophilization, a small amount was vacuum dried and the precise peptides concentration was determined by using Biochrome30 amino acid composition analyzer (Cambridge, UK) as described in [9].

**iTRAQ Labelling**

Five biological replicates from each experimental group were labeled with iTRAQ in accordance with manufacturer’s specifications. Each biological replicate was the result of pooling neutrophil proteins from two rats. A 100 µg of purified peptides from each condition were reconstituted in 20 µL of dissolution buffer and added to the iTRAQ reagent tube already reconstituted in 70 µL of ethanol. The samples were vortexed, spun down and let for 1-2 h at room temperature. To confirm the labeling we usedMALDI and labelled peptides for the three conditions were mixed in 1:1:1 ratio.. Labelling for control, laparotomy and ischemia/reperfusion were 114, 115 and 116 respectively.

**HILIC fractionation of labeled peptides**

Before performing the HILIC fractionation, the iTRAQ labeled peptides were desalted by using in-house packed micro-columns with Poros R2/R3 reversed-phase resin as reported in [10]. HILIC fractionation of the purified labeled peptides was performed [11] and sample replicates were separated into 7 chromatographic fractions. For fractionation, the dried sample was reconstituted in 90% ACN/0.1% TFA and 40 µL of the sample were injected into TSKGel Amide-80 2 mm, 3 μm particle size HPLC column using an Agilent 1200 HPLC system. To elute the peptides a gradient from 90% ACN/0.1% TFA to 60% ACN/0.1% TFA over 35 min awith flow rate of 6 µL/min was used. Fractions were collected automatically at 1 min intervals at UV detection 210nm in a microwell plate and fractions were combined based on their intensities.

**nLC-MS/MS analysis**

The fractionated labeled peptides were analyzed in a Proxeon EASY-nLC system (Thermo Fisher Scientific, Odense, Denmark), connected to an LTQ-Orbitrap Velos (Thermo Scientific) mass spectrometer. The peptides were loaded into an 18 cm in-house packed reversed-phase capillary column (75 µm ID) packed with ReproSil-Pur C18 AQ 3 µm material in solution A0.1% formic acid. Peptides were eluted into the mass spectrometer using 180 min gradients from 0-34% solution-B (95% ACN/0.1% formic acid) with a flow rate of 250 nano-litter/min. Data-dependent acquisition [12] mode was used for MS method whereas to perform a full MS scan mass area of 400-1600 m/z was selected in the Orbitrap with a resolution of 30,000 FWHM (400 m/z) and the target value of 1x10^6^ ions. The top seven intense ions (> 2^+^ charge states) were selected for HCD using a resolution of 7,500 for a complete scan. The HCD settings used were: ion selection threshold was 20000, the maximum injection time for MS/MS was 300 ms, activation time was 0.1 ms, isolation window was 2 m/z, and normalized collision energy was 36.

**Database Searching and Bioinformatics**

Proteome Discoverer version 1.4.0.288 (Thermo Fisher Scientific) was used to process the raw files. to the resultant .mgf files were searched against the UniProt rodent’s database using Mascot (v2.3.2, Matrix Science, London, UK) as a search engine. Following parameters were used to perform the database: fragment (MS/MS) mass tolerance 0.05 Da, precursor mass tolerance 10 ppm, two missed cleavages were allowed and trypsin was selected as a digestion enzyme. Oxidation (M) and deamidation (NQ) were selected as variable modifications whereas Carbamidomethylation of cysteineas a fixed modification. Using Percolator as validator,1% false discovery rate (FDR) was used as a cutoff to filter the identified list of proteins[13].

The mass spectrometry proteomics data were deposited to the ProteomeXchange Consortium via the MassIVE partner repository [14] with the dataset identifier PXD010616.

For more in depth analysis of the regulated proteins, statistics was performed by using R as a statistical platform (http://www.r-project.org/). The expression values from iTRAQ reporter ions for each fraction were log-transformed and median-normalized. Multiple measurements of peptides (with a minimum of two peptides per protein) were combined by the mean intensity in RRollup function of DanteR package [15] and converted into protein quantitation. More sophisticated tools are required to evaluate the statistically significant regulations than just applying standard t-test. The Limma test [3] and rank products [4] are more powerful in dealing with low replicate numbers and higher percentage of missing values [16]. Both tests were applied on all protein ratios using label 114 as a control and corrected for multiple testing error [17]. Proteins with q-value less than 0.05 (5% FDR) were considered significantly regulated.

Cluster analysis was carried out by calculating the mean over all 5 replicated values of each condition. Proteins were merged into one data set. To assign the proteins according to their expression profile in the best number of clusters, two validation indices, Xie-Beni index [1] and minimal centroid distance [2], were used. After determining the fuzzifier parameter, fuzzy c-means clustering [18, 19] was applied and the number of clusters were obtained according to Schwämmle [2]. A standard principal component analysis (PCA) was performed to check similarity and variability among the biological replicates of the same group and the different conditions.

GO *Slim* annotations were obtain from ProteinCenter (Thermo Scientific, Waltham, USA) by applying 5% False discovery rate (FDR). Information to predict the enzyme activity for the identified proteins were retrieved from UniProt database (http://www.uniprot.org/) whereas to put the regulated entities in their respective functional categories like KEGG and Wiki pathways, WebGestalt was used as a platform with default parameters using *Rattus norvegicus* genome as reference set for enrichment analysis [20].

**Evaluation of ROS production and phagocytosis**

Due to the results obtained, neutrophils isolated from the blood of nine more Wistar males submitted to the procedures previously described (grouping, surgical procedures and neutrophil isolation) were tested for ROS production by incubation of 3,2x10^5^ cells with Nitroblue Tetrazolium (NBT) solution for 20min at 37°C. An aliquot of 6 uL was taken from each sample and put on glass slides, fixed with methanol, counterstained with safranin and washed for optical microscopy analysis. Neutrophils were evaluated for activation pattern (intensity and distribution of formazan crystals) and 300 cells were per replicate were counted and evaluated for activation.

Cells from the same animals were incubated with *Saccharomyces cerevisiae* for 30 minutes at 37ºC in a ratio of 3,2x10^5^ neutrophils to 2.5x10^5^ yeast cells. Right after incubation, an aliquot of 6uL was taken from each sample and was fixed on glass slides, stained with panoptical stain, and analyzed by optical microscopy. Neutrophils were evaluated for morphology and counted for phagocytosis. Significance of the differences was evaluated by ANOVA followed by Tukey test.

**References**

1. Xie, X.L.L. and G. Beni, *A Validity Measure for Fuzzy Clustering.* Ieee Transactions on Pattern Analysis and Machine Intelligence, 1991. **13**(8): p. 841-847.

2. Schwammle, V. and O.N. Jensen, *A simple and fast method to determine the parameters for fuzzy c-means cluster analysis.* Bioinformatics, 2010. **26**(22): p. 2841-8.

3. Smyth, G.K., *Limma: linear models for microarray data.*, in *Bioinformatics and Computational Biology Solutions using R and Bioconductor*. 2005, Springer: New York.

4. Breitling, R., et al., *Rank products: a simple, yet powerful, new method to detect differentially regulated genes in replicated microarray experiments.* FEBS Lett, 2004. **573**(1-3): p. 83-92.

5. Tahir, M., et al., *Evaluation of the effects of ischemic preconditioning on the hematological parameters of rats subjected to intestinal ischemia and reperfusion.* Clinics (Sao Paulo), 2015. **70**(1): p. 61-8.

6. E. M. S, R.-C., et al., *Comparative Study of Four Isolation Procedures to Obtain Rat Neutrophils.* Comparative Clinical Pathology, 2014. **11**: p. 71-76.

7. Wisniewski, J.R., et al., *Universal sample preparation method for proteome analysis.* Nat Methods, 2009. **6**(5): p. 359-62.

8. Leon, I.R., et al., *Quantitative assessment of in-solution digestion efficiency identifies optimal protocols for unbiased protein analysis.* Mol Cell Proteomics, 2013. **12**(10): p. 2992-3005.

9. Laursen, I., et al., *Characterisation of the 1st SSI purified MBL standard.* Clin Chim Acta, 2008. **395**(1-2): p. 159-61.

10. Melo-Braga, M.N., et al., *Comprehensive quantitative comparison of the membrane proteome, phosphoproteome, and sialiome of human embryonic and neural stem cells.* Mol Cell Proteomics, 2014. **13**(1): p. 311-28.

11. McNulty, D.E. and R.S. Annan, *Hydrophilic interaction chromatography reduces the complexity of the phosphoproteome and improves global phosphopeptide isolation and detection.* Mol Cell Proteomics, 2008. **7**(5): p. 971-80.

12. Meddah, A.T., et al., *The effects of mesenteric ischemia on ileal colonization, intestinal integrity, and bacterial translocation in newborn piglets.* Pediatr Surg Int, 2001. **17**(7): p. 515-20.

13. Spivak, M., et al., *Improvements to the percolator algorithm for Peptide identification from shotgun proteomics data sets.* J Proteome Res, 2009. **8**(7): p. 3737-45.

14. Vizcaino, J.A., et al., *ProteomeXchange provides globally coordinated proteomics data submission and dissemination.* Nat Biotechnol, 2014. **32**(3): p. 223-6.

15. Taverner, T., et al., *DanteR: an extensible R-based tool for quantitative analysis of -omics data.* Bioinformatics, 2012. **28**(18): p. 2404-6.

16. Schwammle, V., I.R. Leon, and O.N. Jensen, *Assessment and improvement of statistical tools for comparative proteomics analysis of sparse data sets with few experimental replicates.* J Proteome Res, 2013. **12**(9): p. 3874-83.

17. Storey, J.D., *A direct approach to false discovery rates.* J. R. Statist. Soc. B.,, 2002. **64**(3): p. 479-498.

18. Futschik, M.E. and B. Carlisle, *Noise-robust soft clustering of gene expression time-course data.* J Bioinform Comput Biol, 2005. **3**(4): p. 965-88.

19. Bezdek, J.C., *Cluster validity with fuzzy sets.* J. Cybernetics, 1973. **3**(3): p. 58-73.

20. Wang, J., et al., *WEB-based GEne SeT AnaLysis Toolkit (WebGestalt): update 2013.* Nucleic Acids Res, 2013. **41**(Web Server issue): p. W77-83.
