# Supplementary material for: Analysis of the Effect of Intestinal Ischemia and Reperfusion on the Rat Neutrophils Proteome
Source: Front Mol Biosci. 2018 Nov 29;5:89. doi: 10.3389/fmolb.2018.00089 (PMC6281993; doi:10.3389/fmolb.2018.00089)
Supplement: Supplementary file 4 [file Image_1.pdf]

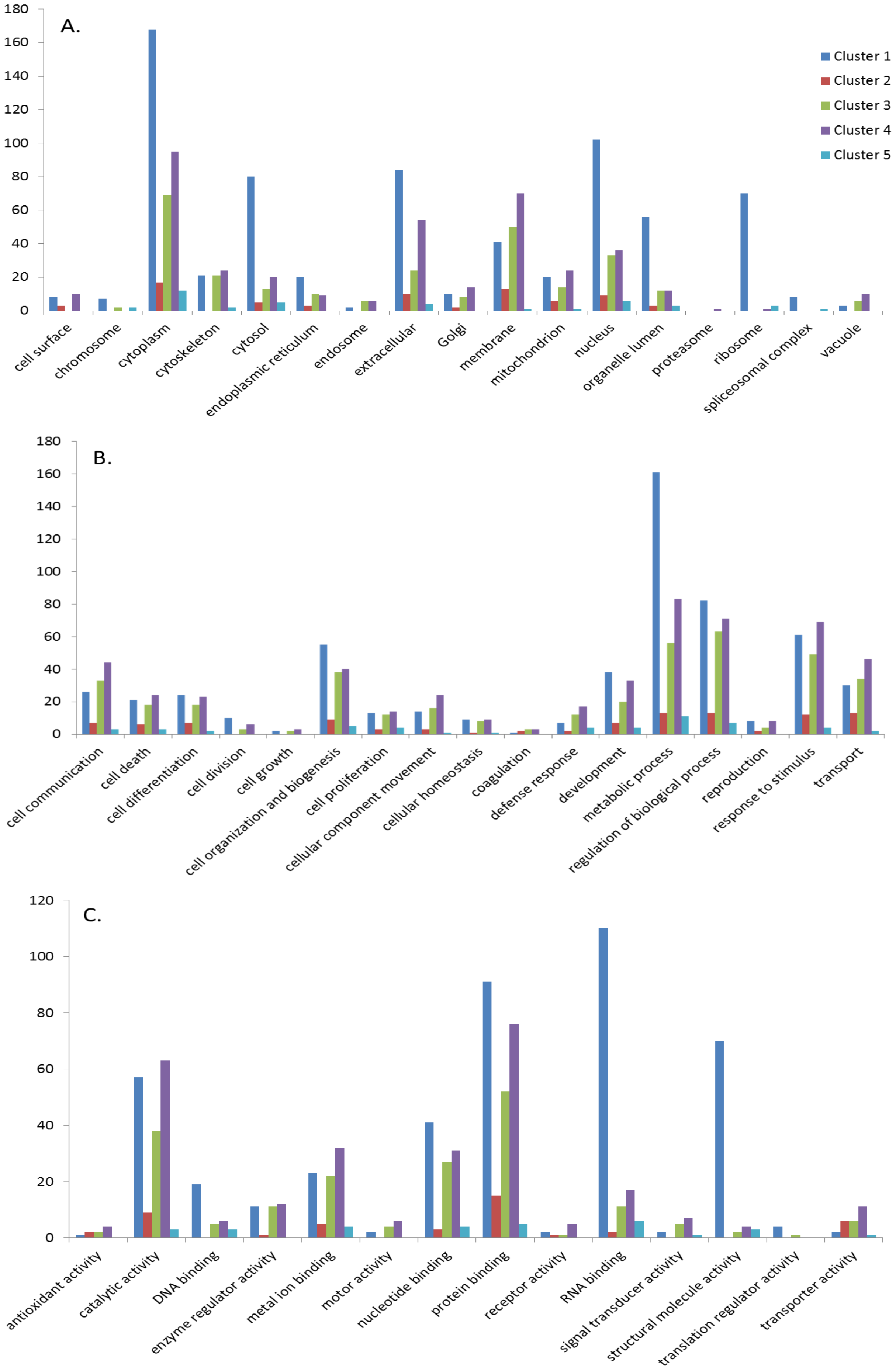

Supplementary figure S1: GO slim analysis fo the identified proteins grouped by abundance cluster. A:Cellular component terms; B: Biological process terms; C: Molecular function terms.
